# Dyslipidaemia and inflammation are risks for subclinical atherosclerotic vascular disease among chronic kidney failure patients in South Africa

**DOI:** 10.1371/journal.pone.0323882

**Published:** 2025-11-10

**Authors:** S. O. Oguntola, M.O. Hassan, R. Duarte, C. Dickens, T. Dix-Peek, T. Snyman, K. Moodley, G. Olorunfemi, A. Vachiat, P. Manga, S. Naicker

**Affiliations:** 1 Division of Nephrology, Department of Medicine, Faculty of Health Sciences, University of Witwatersrand, Johannesburg, South Africa; 2 Nephrology Unit, Department of Medicine, Faculty of Clinical Sciences, College of Medicine and Health Sciences, Afe Babalola University, Ado-Ekiti, Ekiti State, Nigeria; 3 Department of Internal Medicine, Uniosun Teaching Hospital, Osogbo, Osun State, Nigeria; 4 Department of Medicine, Obafemi Awolowo University, Ile-Ife, Nigeria; 5 Department of Internal Medicine Laboratory, Faculty of Health Sciences, University of Witwatersrand, Johannesburg, South Africa; 6 Department of Chemical Pathology, University of the Witwatersrand, Johannesburg, South Africa; 7 Division of Epidemiology and Biostatistics, School of Public Health, University of the Witwatersrand, Johannesburg, South Africa; 8 Department of Medicine, Division of Cardiology, Faculty of Health Sciences, University of Witwatersrand, Johannesburg, South Africa; Saud Al-Babtain Cardiac Centre, SAUDI ARABIA

## Abstract

**Background:**

Chronic kidney disease (CKD) is associated with generalized inflammation. The presence of CKD-related (non-traditional) cardiovascular disease (CVD) risk factors such as inflammation, oxidative stress and uraemic toxins worsen the CVD. A distinct form of lipoprotein alteration known as uraemic dyslipidaemia, characterized by normal low-density lipoprotein (LDL), reduced high density lipoprotein (HDL) and elevated triglyceride and lipoprotein (a) has been described in CKD. The combination of all these factors increase the cardiovascular risk in CKD patients. We evaluated the relationship of lipoprotein and inflammatory biomarkers to atherosclerotic vascular disease (AsVD) among stage 3 CKD, end stage kidney disease (ESKD) patients on continuous ambulatory peritoneal dialysis (CAPD) and haemodialysis (HD) and kidney transplant recipients (KTRs).

**Methods:**

This was a cross-sectional study of 40 adult (18–65 years) non-diabetic stage 3 CKD patients, 40 CAPD and 40 HD patients, 41 KTRs and 41 age- and sex-matched healthy controls. Socio-demographic and cardiovascular risk factors were documented and serum samples were analysed for inflammatory and lipoprotein markers. Echocardiography was performed and carotid intima media thickness (CIMT) was measured in all participants.

**Results:**

The overall prevalence of AsVD was 52.8% in the study population, with the highest burden of inflammation present in CAPD patients. Significantly increased levels of hsCRP, pentraxin-3, Lp(a) and Lp-PLA2 were seen in CAPD, compared to controls. Older age, male gender, reduced high-density lipoprotein (HDL-C) and elevated Lp(a) levels were independently associated with AsVD.

**Conclusion:**

The burden of inflammation and lipoprotein abnormalities was greatest among end stage kidney disease (ESKD) patients and was the highest in CAPD patients. Lipoprotein(a) independently predicted AsVD.

## Introduction

The prevalence of cardiovascular disease (CVD) among chronic kidney disease (CKD) patients was more than five times higher than in patients without CKD (38.4% vs 7.1%) [1]. Inflammation, indicated by elevated high sensitivity C-reactive protein (hsCRP), an acute phase reactant that belongs to the short pentraxin arm of the pentraxin superfamily, has been associated with CKD and CVD and has been suggested to provide prognostic information in CKD patients [2]. However, there are some inconsistencies in the reported association between hsCRP and CKD, with some studies demonstrating significantly elevated levels of other inflammatory markers such as tumour necrosis factor alpha (TNF-α), but not high sensitivity C-reactive protein (hsCRP), in CKD [3,4]. Pentraxin-3, the prototypical example of the long pentraxin arm of the pentraxin superfamily has been shown to perform a vital regulatory role in innate immunity and inflammation [5,6]. Local expression of pentraxin-3 by inflammatory and endothelial cells at the site of inflammation and atherosclerosis, in response to diverse stimuli including TNF-α as opposed to systemic production of CRP from the liver, suggests that pentraxin-3 could have a strong association with atherosclerosis [6]. Accordingly, pentraxin-3 was shown to be a better predictor of CVD than hsCRP in maintenance haemodialysis (HD) patients [7].

Dyslipidaemia is a traditional risk factor for CVD that is more prominent in CKD patients due to the characteristic alteration in lipoprotein moieties [8]. Lipoprotein(a) [Lp(a)] is a cholesteryl-ester-rich macromolecular complex secreted by the liver [9]. Elevated levels of Lp(a) have been associated with myocardial infarction and mortality among patients with CKD [10]. The atherogenic risk of low density lipoprotein (LDL-C) and Lp(a) was studied among patients with myocardial infarction, and Lp(a) was reported to be ten times more atherogenic than LDL-C [11]. Lipoprotein(a) has been described as a cardiovascular risk factor and increased levels have been reported among African Americans [12]. Lipoprotein-associated phospholipase A2 (Lp-PLA2), also known as platelet activating factor acetyl hydrolase (PAF-AH), is expressed by cells critical for the formation of atherosclerotic plaques such as macrophages, T-lymphocytes and mast cells [13]. Lipoprotein-associated phospholipase A2 circulates in the blood mainly bound to LDL-C, with about 66% bound to LDL-C and the remainder bound to high- density lipoprotein (HDL-C and very- low- density lipoprotein (VLDL-C) [14]. The Atherosclerotic Risk in Community (ARIC) Study reported Lp-PLA2 as an independent predictor of coronary heart disease among patients with low LDL-C [15]. Among the traditional lipid profile parameters, HDL-C is unique because it is involved in reverse cholesterol transport (RCT) with anti-atherogenic properties [16]. We evaluated the relationship of dyslipidaemia and inflammation to AsVD among stage 3 CKD, end stage kidney disease (ESKD) patients on continuous ambulatory peritoneal dialysis (CAPD), haemodialysis (HD) and kidney transplant recipients (KTRs).

## Methods

This was a cross-sectional study of 40 adult (age 18–65 years) non-diabetic stage 3 CKD patients, 40 patients on HD, 40 patients on CAPD, 41 KTRs and 41 age- and sex-matched healthy controls at a large urban public hospital in South Africa from 2 January 2017–31 August 2017. The study was approved by the Human Research Ethics Committee of the University of Witwatersrand (study number M160614) and all procedures were performed in accordance with relevant guidelines and regulations.

The CAPD patients were on 4 exchanges/day using 2 L bags of conventional (glucose-based) CAPD fluid, while the HD patients were on thrice weekly maintenance dialysis using high flux dialyzers with biocompatible membranes, bicarbonate dialysate and moderate blood flow rate of ≥300 ml/min. An interviewer-administered questionnaire was used to obtain information on participants’ socio-demographic and cardiovascular risk factors. With patients in an erect position, waist and hip circumference were measured; this was done after draining out PD fluid in CAPD patients, and the waist-hip ratio was then calculated. In HD patients, the post-HD weight was used in the analysis; weight was obtained in CAPD patients after the PD fluid was drained. Body mass index (BMI) was calculated using the formula weight/height^2^, while the body surface area was calculated using the Mosteller formula [17]. Three blood pressure readings were taken on the left arm with an appropriate-sized cuff, 10 minutes apart, after the patient had rested for 5 minutes in a sitting position. The average of the last two readings was recorded as the blood pressure. Blood samples were taken before dialysis in HD patients and serum lipids levels were determined by an enzymatic colorimetric method using Roche Cobas 8000 modular c701 analyzer (Roche, Japan). The serum levels of inflammatory markers [pentraxin-3, hsCRP, TNF-α and the ligand of the receptor for advanced glycation end-products (EN-RAGE)] were determined by Magnetic Luminex Assay, using a Human Premixed Multi-analyte kit (R&D Systems, Inc. USA) using a BioPlex 200 System (Bio-Rad Laboratories Inc., USA). High sensitivity C-reactive protein levels were measured using HycultBiotech human HK369 Elisa kit (HycultBiotech, Netherlands). Lipid biomarkers [Apolipoprotein A1 (Apo A1) and Lp-PLA2] were determined by Magnetic Luminex Assay, Human Premixed Multi-Analyte Kit (R&D Systems, Inc., USA) using BioPlex 200 Systems (Bio-Rad Laboratories Inc., USA). Lipoprotein(a) assay was determined using Beckman Immage® 800 Immunochemistry system (Beckman Coulter, USA). Echocardiography was performed on all patients and carotid intima media thickness (CIMT) was assessed. Echocardiography and CIMT were performed in CAPD patients after draining out the PD fluid and before initiation of dialysis in HD patients. Echocardiography and CIMT measurements were performed in accordance with the guidelines of the American Society of Echocardiography [18], using a Philips iE33 echocardiography machine (Philips Corporation, USA). Carotid intima media thickness was assessed using the vascular probe of the echocardiography machine, Philips iE33 (S5-1 probe) by focussing on the far wall of the carotid artery, 1 cm proximal to the dilatation of the carotid bulb along the long axis of the artery. Automatic echo-generated measurements with percentage quality of 95% were recorded. The procedure was carried out on both left and right carotid arteries and the average used in the analysis. Atherosclerotic vascular disease was defined by CIMT (> 0.55 mm).

### Data analysis

Stata version13.1 (Stata Corp LP, College Station, TX, USA) and IBM SPSS version 20 (IBM Corporation, Armonk, New York) were used for statistical analysis. Categorical variables were expressed as frequencies and percentages and tested using the chi-square test. A test of normality (Shapiro-Wilk) was performed on all continuous variables and data was presented as median and interquartile ranges (IQR). Comparisons were performed between the CKD groups and controls using the Wilcoxon rank-sum test; further comparisons were done between CKD, CAPD, HD, KTRs and controls using the Kruskal-Wallis test, and the post-hoc Dunn’s test. Comparisons were made between patients who had AsVD and patients who did not have AsVD using the Wilcoxon-rank sum test. Univariable logistic regression analyses were performed, followed by multivariable binary logistic regression analysis to determine predictors of AsVD. The multivariable model was built in a stepwise backward elimination technique using explanatory variables with univariable p-value < 0.2. Some variables such as BMI, calcium-phosphate product, pentraxin-3, hsCRP, TNF-α and Apo A1 were chosen *a priori* based on literature search and experience of the researchers. Multicollinearity was checked using variance inflation factor (VIF).

### Sample size calculation

Using STATA version 13, the sample size was calculated based on the difference in atherosclerosis and biomarkers of inflammation.

The power to detect differences in levels of atherosclerosis was calculated. With sample size of 40 in each group, a 5% significance level and atherosclerosis proportion of 38.1% in hemodialysis patients [19] compared to 7.93% in controls,^19^ there will be a power of 90.89 to detect differences in these group.

With sample size of 40 in each group, a 5% significance level and elevated Lp(a) proportion of 70% in CAPD patients compared to 15% in controls, there will be a power of 99.8 to detect differences in these group [20].

With the sample size of 40 in each group, a 5% significance level and CRP mean of 8.06 ± 1.31 in haemodialysis patients compared to 2.27 ± 0.34 in healthy controls,^29^ there will be a power close to 100 to detect differences in these group [19].

## Results

There was a similar age distribution across the groups; [41 years (IQR = 36.0–51.5) among stage 3 CKD patients, 39.5 years (IQR = 35.0–46.5) among CAPD patients, 40.5 years (IQR = 36.0–49.0) among HD patients, 39 years (IQR = 30.0–52.0) among KTRs and 41.0 years (29.0–48.0) among controls], Table 1. There were 78 females and 83 males in the study population. The median duration on CAPD and HD was similar [3.0 years (IQR: 1.5–4.0) in CAPD patients and 3.0 years (IQR: 2.0–6.0) in HD patients], while the median post-transplant duration was 4 years (IQR: 1–7). Significantly increased systolic blood pressure (SBP), diastolic blood pressure (DBP) and mean arterial blood pressure (MABP) were seen in all the kidney disease groups (CKD, CAPD, HD and KTRs) compared to controls; Table 1. Among CAPD patients, significantly increased levels of total cholesterol (TC) were present compared to controls. Serum albumin levels were significantly reduced in all CKD groups except KTRs. Statin use was most frequent among KTRs (51.2%, 21/41); The levels of serum phosphate and calcium-phosphate products were significantly increased among CAPD patients compared to control and KTRs. Eighty percent of ESKD patients were on phosphate binders comprising calcium carbonate. Pentraxin-3 levels were significantly increased in all the study groups (CKD stage 3, PD, HD, KTRs) compared to controls while EN-RAGE was only significantly increased among KTRs compared to controls. A marked increase in the levels of Lp(a) and TNF-α were observed in CAPD patients. Lp(a) was significantly increased among CAPD patients compared to controls. The levels of Lp(a) in CAPD patients was more than double the levels in HD patients; Table 1.

**Table 1:** Sociodemographic and clinical characteristics of the study population.

Parameter^#^	Control (n = 41)	CAPD (n = 40) p-value		HD (n = 40) p-value		CKD STAGE 3 (n = 40) p-value		KTRs (n = 41) p-value	
Age	41 (29–48)	39.5 (35–46.5)	0.561	40.5 (36.0–49)	0.564	41 (36–51.5)	0.323	39 (30–52)	0.824
WHR	0.85 (0.82–0.89)	0.92 (0.87–0.96)	<0.001*	0.9 (0.87–0.94)	0.002*	0.92 (0.84–0.99)	0.003*	0.89 (0.86–0.94)	0.003*
SBP	125 (119–132)	147 (127–162)	<0.001*	155 (138–171)	<0.001*	138 (125–156)	<0.001*	139 (128–151)	<0.001*
Creatinine (µmol/l)	80 (63–89)					124 (106–166)	<0.001*	123 (91–152)	<0.001*
Albumin (mmol/l)	44.0 (42–45)	35.5 (33.0–40.0)	<0.001*	38.5 (35.0–40.0)	<0.001*	41.5 (38.5–44.0)	0.014*	43.0 (42.0–48.0)	0.505
Calcium (mmol/l)	2.33 (2.28–2.45)	2.29 (2.12–2.40)	0.025*	2.29 (2.15–2.40	0.088	2.34 (2.27–2.44)	0.928	2.44 (2.34–2.59)	0.004*
Phosphate (mmol/l)	1.0 (0.9–1.1)	1.6 (1.2–1.9)	<0.001*	1.2 (0.8–1.4)	0.073	1.1 (0.9–1.2)	0.052	0.97 (0.85–1.07)	0.516
CaXPO_4_ (mg^2^/dl^2^)	29.5 (26.7–32.5)	40.1 (32.1–53.7)	<0.001*	35.4 (22.4–41.0)	0.228	31.4 (26.5–37.5)	0.132	29.8 (22.9–35.9)	0.756
TC (mmol/l)	4.3 (3.7–4.7)	5.2 (4.2–6.1)	0.001*	3.4 (3.0–3.9)	<0.001*	4.4 (4.1–5.1)	0.189	4.46 (3.93–4.93)	0.330
TG (mmol/l)	1.3 (0.8–1.5)	1.3 (1.0–2.0)	0.364	0.9 (0.6–1.2)	0.018*	1.3 (1.0–1.9)	0.511	1.3 (1.1–1.9)	0.399
LDL-C (mmol/l)	2.6 (2.1–3.2)	3.0 (2.4–4.1)	0.062	1.8 (1.4–2.3)	<0.001*	2.7 (1.9–3.1)	0.660	2.6 (2.2–3.1)	0.777
HDL-C (mmol/l)	1.2 (1.0–1.5)	1.2 (0.9–1.4)	0.872	1.0 (0.9–1.3)	0.051	1.3 (1.1–1.5)	0.296	1.3 (1.1–1.5)	0.467
Apo A1 (mg/dl)	14.5 (12.3–17.3)	13.5 (10.1–15.4)	0.078	12.1 (10.2-15.6)	0.063	13.9 (11.7-18.1)	0.951	13.4(10.5 - 16.3)	0.905
Lp-PLA2 (ng/ml)	89.8 (50.9–139.2)	158.4(77.3 204.5)	0.007*	129.3(71.5-187.6)	0.060	110.2(73.9-153.3)	0.225	114.9(66.7-171.6)	0.141
Lp(a) (mg/dl)	40.2 (29.3–50.4)	123 (80.8-173.0)	<0.001*	55.1 (41.8-105.5)	0.019*	65.3 (29.1 - 99.9)	0.056	69.3(36.4–125)	0.068
hsCRP (mg/l)	1.5 (0.6–4.5)	7.4(2.7–13.0)	<0.001*	6.7 (3.0–12.3)	<0.001*	4.4 (1.5–11.7)	0.013*	2.5 (1.0–6.4)	0.239
Pentraxin-3 (ng/ml)	1.1 (0.4–1.9)	3.4 (1.6–5.7)	<0.001*	1.9 (1.1–3.4)	0.009*	2.8 (0.7–4.1)	0.009*	1.6 (0.9–2.7)	0.023*
TNF-alpha (pg/ml)	2.5 (0.5–26.9)	29.1 (7.0–78.3)	<0.001*	4.0 (0.8–20.7)	0.576	4.0 (2.1–26.7)	0.309	14.1 (3.3–26.3)	0.083
EN-RAGE (ng/ml)	36.5 (16.7–88.9)	61 (28.8–110.6)	0.139	34.6 (17.0–66.2)	0.491	42.5 (30.0–70.9)	0.590	91.1 (33.2–260.8)	0.004*
CIMT (mm)	0.49 (0.46–0.52)	0.63 (0.55–0.71)	<0.001*	0.55 (0.50–0.62)	<0.001*	0.55 (0.48–0.61)	<0.001*	0.55 (0.48–0.63)	0.001

# median (interquartile range), Wilcoxon rank-sum test; ^*^ p < 0.05 was significant, p values were obtained from comparison of each CKD groups (CAPD, HD, CKD Stage 3, KTRs) with controls; ESKD – end-stage kidney disease; CAPD – continuous ambulatory peritoneal dialysis; HD – haemodialysis; KTRs – kidney transplant recipients; WHR – waist hip ratio; SBP – systolic blood pressure; DOD/PTD – duration on dialysis and post-transplant duration; eGFR – estimated glomerular filtration rate; TC – total cholesterol; LDL-C – low density lipoprotein; TG – triglyceride; HDL-C – high density lipoprotein; Apo A1 – apolipoprotein A1; Lp-PLA2 – lipoprotein-associated phospholipase A2; Lp(a) – lipoprotein(a); TNF-α – tumour necrosis factor-alpha; EN-RAGE – ligand for receptor of advance glycation end product; CIMT – carotid intima media thickness

When the five groups were compared, all inflammatory and lipoprotein biomarkers (except ApoA1) showed significant increase in at least one of the comparator groups, evidenced by significance with Kruskal-Wallis and Dunn’s tests. The difference was strongest when CAPD patients were compared with controls; Table 2.

**Table 2:** Clinical, laboratory and echocardiographic parameters in CKD, HD, CAPD patients, kidney transplant recipients and controls.

Parameter	K-W test (n = 202) χ ^2^ (p-value)*	Dunn test (p-value)*						
		CKD/ Controls	CAPD/ Controls	HD/ controls	KTRs/ Controls	CKD/ CAPD	CKD/ HD	KTRs/ CAPD
Age (years)	1.32 (0.858)							
BMI (Kg/m^2^)	19.6 (0.001)	0.597	0.132	0.468	0.162	0.008	0.001	0.908
WHR	21.6 (<0.001)	0.001	<0.001	0.031	0.064	0.758	0.310	0.164
SBP (mmHg)	42.9 (<0.001)	0.002	<0.001	<0.001	0.004	0.214	0.154	0.142
DBP (mmHg)	33.5 (<0.001)	0.018	<0.001	<0.001	0.009	0.043	0.088	0.065
MABP (mmHg)	129.1 (<0.001)	<0.001	<0.001	<0.001	0.295	0.183	0.095	<0.001
TC (mmol/l)	47.2 (<0.001)	0.931	0.272	<0.001	0.646	0.007	<0.001	0.798
LDL-C (mmol/l)	35.9 (<0.001)	0.333	0.277	<0.001	0.700	0.041	0.004	0.142
HDL-C (mmol/l)	11.9 (0.018)	0.462	0.212	0.037	0.260	0.611	0.182	0.018
eGFR (ml/min/1.73m^2^)	171.1 (<0.001)	<0.001			0.029			
Calcium(mmol/l)	34.8 (<0.001)	0.195	0.195	0.743	0.002	0.999	0.345	<0.001
Phosphate(mmol/l)	34.9 (<0.001)	0.261	<0.001	0.061	0.487	<0.001	0.458	<0.001
CaXPO_4_(mg^2^/dl^2^)	25.9 (<0.001)	0.203	<0.001	0.128	0.832	0.013	0.805	<0.001
Albumin(g/l)	61.8 (<0.001)	0.326	<0.001	<0.001	0.831	<0.001	0.150	<0.001
hsCRP (mg/l)	23.2 (<0.001)	0.129	<0.001	0.004	0.299	0.117	0.288	0.024
Pentraxin-3 (ng/ml)	22.8 (<0.001)	0.059	<0.001	0.361	0.452	0.525	0.515	0.067
ENRAGE (ng/ml)	16.8 (0.002)	0.688	0.128	0.473	0.027	0.266	0.266	0.146
TNF-α (ng/ml)	25.2 (<0.001)	0.419	<0.001	0.784	0.571	0.004	0.595	0.127
Lp(a) (mg/dl)	27.7 (<0.001)	0.832	<0.001	0.293	0.780	0.007	0.657	0.007
LP-PLA2 (mg/ml)	12.4 (0.015)	0.290	0.046	0.053	0.977	0.077	0.382	0.042
Apo A1	6.8 (0.137)							
EF (%)	62.6 (<0.001)	<0.001	<0.001	0.005	0.168	0.413	0.131	<0.001
LVMI	25.6 (<0.001)	0.222	0.013	0.038	<0.001	0.463	0.967	0.186

*p < 0.05 was significant, Kruskal-Wallis and Dunn’s tests; CKD- chronic kidney disease; HD- haemodialysis; CAPD- continuous ambulatory peritoneal dialysis; K-W- Kruskal Wallis; BMI – body mass index; WHR – waist-hip ratio; SBP – systolic blood pressure; MABP – mean arterial blood pressure; TC – total cholesterol; TG – triglyceride; LDL – low density lipoprotein; HDL – high density lipoprotein; eGFR – estimated glomerular filtration rate; hsCRP – high sensitivity C reactive protein; ENRAGE – the ligand of the receptor for advanced glycation end-products; TNF-α – tumour necrosis factor alpha; Lp(a) – lipoprotein(a); Lp-PPLA2 – lipoprotein-associated phospholipase A2; Apo A1 – Apolipoprotein A1; EF – ejection fraction; LVMI – left ventricular mass index

The overall prevalence of AsVD in the study population was 52.8%. The patients who had AsVD were significantly older than patients who did not have AsVD; Table 3. Significantly increased WHR and LVMI were seen among patients with AsVD. Lipoprotein(a) levels were increased in patients who had AsVD compared to patients who did not have AsVD, while HDL-C levels were significantly decreased in patients with AsVD.

**Table 3:** Risk factors for atherosclerotic vascular disease in the study population.

Parameters	AsVD present N = 85	AsVD absent N = 76	p-value
Age (years)	44.0 (38.0–52.0)	37 (27–43)	<0.001*
Gender			
Female	30.0 (38.5)	48.0 (61.5)	<0.001*^#^
Male	55.0 (66.3)	28.0 (33.7)	
BMI (Kg/m^2^)	27.0 (22.5–31.9)	26.4 (22.0–30.5)	0.394
WHR	0.92 (0.88–0.96)	0.89 (0.85–0.93)	0.003*
SBP (mmHg)	145.0 (130.0–160.0)	145.5 (126.5–158.0)	0.428
TC (mmol/l)	4.3 (3.5–5.1)	4.2 (3.5–5.1)	0.806
TG (mmol/l)	1.3 (0.8–1.7)	1.2 (0.9–1.8)	0.792
LDL-C (mmol/l)	2.4 (1.9–3.1)	2.4 (1.8–3.0)	0.466
HDL-C (mmol/l)	1.1 (0.9–1.4)	1.3 (1.1–1.4)	0.010*
SCr (µmol/l)	456 (124–1018)	211 (126–236)	0.116
Calcium (mmol/l)	2.3 (2.2–2.4)	2.4 (2.2–2.5)	0.309
Phosphate (mmol/l)	1.2 (0.9–1.4)	1.1 (0.9–1.3)	0.218
CaXPO_4_ (mg^2^/dl^2^)	35.5 (26.5–41.4)	31.8 (26.9–38.4)	0.356
Albumin (g/l)	40.0 (35.0–43.0)	40.0 (36.0–44.0)	0.184
hsCRP (mg/l)	5.7 (1.5–11.8)	4.2 (1.5–12.4)	0.473
Pentraxin-3 (ng/ml)	2.3 (0.9–2.6)	2.4 (1.1–4.2)	0.767
TNF-α (pg/ml)	9.1 (3.3–33.1)	7.6 (2.0–33.6)	0.457
ENRAGE (ng/ml)	46.2 (25.9–99.4)	54.7 (28.6–100.7)	0.480
Lp(a) (mg/dl)	84.5 (44.0–140.0)	59.5 (32.6–111.0)	0.091
Lp-PLA2 (ng/ml)	129.8 (61.6–179.8)	109.4 (69.9–162.2)	0.385
ApoA1(g/l)	13.1 (10.5–15.8)	13.6 (10.9–16.7)	0.642
EF (%)	60.0 (55.0–67.0)	63.0 (58.0–68.0)	0.219
LVMI (g/m^2^)	119.6 (96.2–145.5)	105.0 (83.2–131.4)	0.045*

# frequency (percentage), Pearson Chi-squared test, all other results were presented as Wilcoxon rank-sum test, median (interquartile range); ^*^ p < 0.05 was significant; AsVD – atherosclerotic vascular disease; BMI – body mass index; WHR – waist-hip ratio; SBP – systolic blood pressure; TC – total cholesterol; TG – triglyceride; LDL-C – low density lipoprotein; HDL-C – high density lipoprotein; CaXPO_4_ – Calcium-Phosphate product; hsCRP – high sensitivity C reactive protein; EN-RAGE – the ligand of the receptor for advanced glycation end-products; TNF-α – tumour necrosis factor alpha; Lp(a) – lipoprotein(a); Lp-PLA2 – lipoprotein-associated phospholipase A2; ApoA1 – Apolipoprotein A1; EF – ejection fraction; LVMI – left ventricular mass index.

Age, HDL-C, Lp(a) levels and male gender were independent predictors of AsVD when the multivariable logistic regression model was adjusted for age, gender, BMI, calcium-phosphate product (CaXPO_4_), HDL-C, albumin, hs-CRP, Lp-PLA2, ApoA1, TNF-α and Pentraxin-3; Table 4. Thus, the odds of AsVD increased by 7% for an annual increase in age (adjOR: 1.07, 95%CI: 1.04–1.11, P-value < 0.001). The odds of developing AsVD was 3.78-fold among males as compared to female participants (adjOR: 3.78, 95%CI: 1.69–8.44, P-value < 0.001). The odds of having AsVD was 2.15 for every unit increase in Lp(a) (adjOR: 2.15, 95%CI: 2.15, P-value = 0.04).

**Table 4:** Logistic regression showing predictors of atherosclerotic vascular disease.

Parameters	Unadjusted			AdjOR^ɣ^		
	OR	95% CI	p-value	OR	95% CI	p-value
Age^a^	1.07	1.04–1.11	<0.001*	1.07	1.04–1.11	<0.001*
Male Gender	2.98	1.57–5.66	0.001*	3.78	1.69–8.44	0.001*
BMI (≥30 Kg/m^2^)	1.56	0.77–3.17	0.217	1.51	0.63–3.60	0.355
CaXPO_4_ (≥55 mg^2^/dl^2^)	0.86	0.32–2.29	0.758	0.63	0.19–2.07	0.444
Low HDL-C^#^	1.87	1.00–3.51	0.052	2.96	1.35–6.46	0.007*
Albumin (≥35 mmol/l)	0.66	0.38–1.17	0.159	1.06	0.48–2.35	0.893
Pentraxin-3 (≥1.87ng/ml)	0.99	0.53–1.85	0.981	0.84	0.38–1.86	0.663
hsCRP (≥ 4.5 mg/l)	1.38	0.74–2.56	0.313	1.21	0.55–2.63	0.636
TNF-α (≥26.87pg/ml)	1.13	0.61–2.10	0.705	1.64	0.73–3.65	0.229
Lp(a) (>65.4md/dl)	1.97	1.05–3.70	0.034*	2.15	1.02–4.53	0.044*
ApoA1 (<12.3g/l)	0.80	0.43–1.48	0.474	0.62	0.29–1.30	0.205
Lp-PLA2 (>139.2ng/ml)	1.52	0.82–2.84	0.185	1.35	0.62–2.92	0.447

*statistically significant, p < 0.05; ^a^linear trend; AdjOR – adjusted odds ratio, OR – odds ratio; BMI – body mass index; HDL-C – high density lipoprotein; CaXPO_4_ – Calcium-Phosphate product; hsCRP – high sensitivity C-reactive protein; TNF-α – tumour necrosis factor alpha; Lp(a) – lipoprotein(a); ApoA1 – Apolipoprotein A1; Lp-PLA2 – lipoprotein-associated phospholipase A2; AdjOR^ɣ^ - Adjusted for BMI, CaXPO_4_, Albumin, Pentraxin-3, hsCRP, TNF-α, ApoA1 and LpPLA2; OR – odds ratio; CI – confidence interval, ^#^Low HDL-C = < 1.03 in male and < 1.29 mmol/l in female

## Discussion

Lp(a) independently predicted AsVD in our study population after adjusting for age, gender, serum albumin, calcium-phosphate product, BMI, HDL-C, hs-CRP, Lp-PLA2, ApoA1, TNF-α and Pentraxin-3. This is consistent with findings from a previous study [10]. The association between elevated Lp (a) levels and incident myocardial infarction and mortality was assessed among the participants of the Chronic Renal Insufficiency Cohort (CRIC) study followed up for 7.5 years; the study found that the highest quartile of Lp (a), with baseline levels > 61.3 mg/dl was associated with increased risk of myocardial infarction (HR 1.49; 95% CI – 1.05–2.11) and death (HR 1.28; 95% CI – 1.05–1.57) [10]. Similarly, high plasma levels of Lp(a) accelerated the progression of atherosclerosis among ESKD patients via low-density lipoprotein receptor (LDLr) and membrane-bound CXC chemokine ligand 16 (CXCL16)-associated Lp(a) accumulation in arteries [21]. In addition, the levels of CXC16 and LDLr were elevated among ESKD patients with elevated Lp(a) [21]. Previous studies have demonstrated the role of LDLr and CXC16 in the internalization of Lp(a) into vascular endothelial cells [22,23]. It is therefore conceivable that these Lp(a) transport proteins, similar to Lp(a), are cleared ineffectively from the system and/or may be produced in excess from the liver, in response to massive proteinuria and consequent hypoalbuminaemia in CKD patients, with a resultant increase in levels. Lipoprotein(a)-induced atherosclerosis is mediated via various mechanisms including, but not limited to, structural homology to plasminogen resulting in high-affinity binding to fibrinogen and inhibition of plasminogen activation; pro-inflammatory effects resulting in recruitment of inflammatory cells to site of atherosclerosis; inhibition of TGF-β activation; oxidation of Lp(a); and migration and proliferation of smooth muscle cells and endothelial cells [24].

Other independent predictors of AsVD in our study, other than Lp(a), were advancing age, male gender and reduced HDL-C levels after adjusting for confounders. Increasing age has been identified as a predictor of vascular injury due to decrease in the levels of vasoactive nitric oxide [25]. In a previous study, we demonstrated that age (>40years) was associated with and independently predicted AsVD in KTRs and ESKD respectively [26,27]. The Multi-Ethnic Study of Atherosclerosis (MESA) showed a strong association between reduced HDL and CVD with declining GFR [28]. Current concepts of the cardiovascular risk of HDL-C in CKD patients go beyond reduction in levels of HDL-C. The functional complexity of the HDL-C particle, its role in reverse cholesterol transport and the uraemic environment contribute to cardiovascular risk [29].

Our study confirmed previous findings of elevated Lp(a) and Lp-PLA2 among CKD patients compared to controls, with significant differences seen when CAPD patients were compared with controls. Plasma and Lp (a)-associated Lp-PLA2 activity was evaluated in mild-moderate CKD patients, CAPD and HD patients; all patient groups showed significantly increased Lp(a) levels and Lp-PLA2 activity [30]. There was no association demonstrated between Apolipoprotein A1 and AsVD.

We found significantly higher levels of inflammatory markers among all the CKD groups including KTRs in our study. The totality of these findings indicate that chronic inflammation in CKD starts long before CKD patients become dialysis-requiring, and persists with ESKD and in the post-transplant period. Similar results were reported in cross-sectional studies in HD and PD patients [7,31,32]. Plasma levels of pentraxin-3 were shown to be significantly higher among HD patients when compared with healthy controls; pentraxin-3 levels increased rapidly after a single session of HD [7].

We found significantly increased levels of inflammatory biomarkers in CAPD patients, compared to HD patients. This signifies a higher level of inflammation among our PD patients and can be explained by some PD-related factors; firstly, insertion of the peritoneal catheter and filling of the peritoneal cavity with PD fluid triggers an inflammatory response in the peritoneum [33]. A study demonstrated an increase in the levels of inflammatory markers after initiation of PD; persistent rise in levels of inflammatory markers were seen with increasing duration of exposure to PD [33]. Secondly, persistent exposure of peritoneal mesothelial cells to glucose-based peritoneal dialysis fluid triggers an inflammatory response which can self-persist by continuous activation of inflammatory markers [34,35]. Thirdly, formation of glucose degradation product (GDP) which occurs during heat sterilization of glucose-based PD fluids has been implicated in the morphological changes seen in the peritoneum of patients treated with glucose-based PD fluids [36]. Therefore, the combination of low pH (5.2), high lactate levels (40 mmol/l), glucose-induced high osmolarity (395 mOsm) in the conventional PD fluid precipitates GDP in the conventional glucose-based PD fluid, and may explain the high levels of inflammation among our CAPD patients. In addition, a previous history of peritonitis can worsen an underlying inflammatory response in CAPD patients. Peritonitis triggers an inflammatory response which leads to the release of inflammatory cytokines from inflammatory cells to the site of infection, where pentraxin-3 activates the complement system via the classical pathway [37] and enhances phagocytosis of apoptotic cells by dendritic cells and macrophages [38]. A previous study reported the levels of pentraxin-3 in the peritoneal effluent to be higher among patients on longer PD duration [39]. In addition to the PD-related factors discussed earlier, the significantly elevated hs-CRP among CAPD patients may be due to the decline in Residual Renal Function (RRF) and volume overload [40,41]. Compared to CAPD patients who had normal levels of CRP, a cohort of CAPD patients who had high CRP levels (>10mg/L), after one year on maintenance dialysis, experienced a more rapid decline in RRF [40].

This study is not without limitations. The cross-sectional nature of this study allowed for measurements of the various parameters at a single point; a longitudinal study will provide data on the evolution of atherosclerosis over the period of CKD and dialysis. The small study population in a single centre may also not be generalisable. While the sample size is small, the number was selected based on sample size calculation with adequate power to detect a difference in the groups studied. Residual Renal Function and fluid overload had not been systematically recorded in case files of our cohort.

In conclusion, the burden of inflammation and lipoprotein abnormalities was greatest among ESKD patients, with the highest burden in CAPD patients. Kidney transplant recipients have a more favourable lipid biomarker profile compared to dialysis patients. Advancing age, male gender, low HDL-C and elevated Lp(a) independently predicted AsVD. Early assessment of levels of Lp(a) and HDL-C and commencement of strategies to lower Lp(a) levels and increase HDL-C levels will be beneficial among CKD patients.
